# Fabrication of CuO/BiVO_4_ composites for enhanced visible-light-driven photocatalytic antibacterial activity

**DOI:** 10.1039/d5ra06653k

**Published:** 2025-11-18

**Authors:** Jiao Zhao, He Yang, Chen Chen, Tong You, Xingyi Yu, Yan Zhang, Hongyan Liu, Yimin Zhu

**Affiliations:** a Collaborative Innovation Center for Vessel Pollution Monitoring and Control, Dalian Maritime University Dalian 116026 China zhaojiao@dlmu.edu.cn ntp@dlmu.edu.cn

## Abstract

In recent years, photocatalytic antibacterial technology has attracted wide attention due to its advantages of broad-spectrum antibacterial activity, high stability, safety and non-toxicity. Bismuth vanadate (BiVO_4_) reveals a strong response to visible light, however, the photocatalytic activity of pure BiVO_4_ remains unsatisfactory. In the present work, CuO/BiVO_4_ composites were successfully constructed, and the photocatalytic performance and antibacterial mechanism were systematically studied. The antibacterial results confirmed that the CuO/BiVO_4_ composites exhibited enhanced photocatalytic antibacterial activity against *Escherichia coli* (*E. coli*). With the CuO addition of 12.5%, the CuO/BiVO_4_ composites presented superior antibacterial performance, and the antibacterial rate reached 100% under visible light irradiation for 30 min, while the antibacterial rate for BiVO_4_ was less than 20% under the same conditions. In addition, CuO/BiVO_4_ composites displayed a long-term effect, and the antibacterial rate was kept at >90% after 5 cycles. The antibacterial mechanism was mainly from ROS oxidative damage, in which ·O_2_^−^ played a major role in antibacterial activity.

## Abstract 2

CuO/BiVO_4_ composites were constructed for enhanced photocatalytic antibacterial activity mainly via ROS oxidative damage.

## Graphical abstract

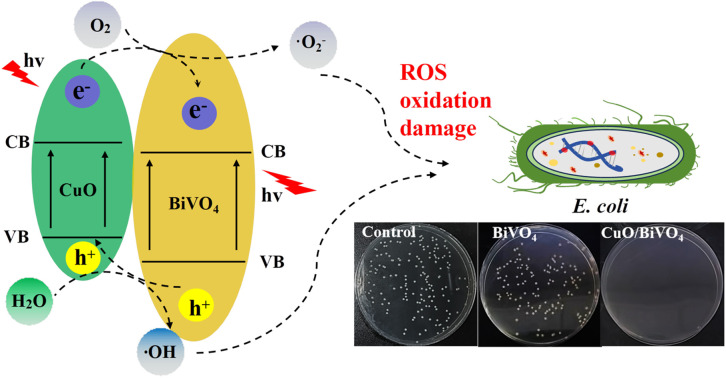

## 1. Introduction

The proliferation of bacteria is becoming increasingly serious and constantly threatening human health and life, and therefore, it is urgent to address the bacterial contamination. Photocatalysis, as an economical, efficient, and environmentally friendly method, has attracted increasing attention in various research fields over the past decades, such as in photocatalytic degradation of pollutants, water splitting, and photocatalytic antibacterial applications. When a photocatalyst absorbs light, it generates e^−^–h^+^ pairs, which react with water and dissolved oxygen to form reactive oxygen species (ROS),^1,2^ such as hydroxyl radicals (·OH), singlet oxygen (^1^O_2_) and superoxide radicals (·O_2_^−^). These highly oxidative reactive species can disinfect pathogens by destroying essential macromolecules within bacteria.^3^ It has been reported that TiO_2_ exhibits photocatalytic activity against several types of microorganisms, including bacteria and viruses.^4^ However, TiO_2_ only harvests ultraviolet light (*L* <390 nm, accounting for only 4% of total sunlight) because of the large bandgap energy.^5,6^

BiVO_4_ is a non-toxic, corrosion-resistant, highly stable, and environmentally friendly photocatalyst with significant application prospects. Since Kudo *et al.* first reported the photocatalytic water splitting of BiVO_4_ under visible light in 1998,^7^ it has attracted much attention in the fields of photocatalytic degradation of organic pollutants, hydrogen evolution from water splitting, and antibacterial applications.^8–14^ There are three different crystal phases for BiVO_4_, tetragonal zircon (t-z), tetragonal scheelite (t-S), and monoclinic scheelite (m-S).^15,16^ Given that the monoclinic scheelite phase (m-BiVO_4_) is the most thermodynamically stable and has demonstrated the highest photocatalytic activity (*e.g.*, for visible-light degradation of pollutants and water splitting), it has attracted widespread research interest. Consequently, we focus herein on the crystal structure, electronic structure, and optical characteristics of m-BiVO_4_ to elucidate their influence on its photoelectrochemical properties. The conduction band of monoclinic BiVO_4_ is mainly composed of V 3d orbitals, and the valence band is a hybrid of Bi 6*s* and O 2s orbitals. The hybrid orbitals can reduce the bandgap, making BiVO_4_ broader visible light absorption region and suitable energy level positions as a visible-light-driven photocatalyst. However, the rapid recombination of photogenerated charge carriers and poor surface adsorption capacity greatly limits the practical application of pure BiVO_4_.^17–19^

In order to improve the photocatalytic performance, heterojunctions have been constructed, such as Cu/BiVO_4_,^20^ Ag/BiVO_4_,^21^ WO_3_/BiVO_4_,^22^ V_2_O_5_/BiVO_4_,^23^ CeO_2_/BiVO_4_,^24^ Bi_2_O_3_/BiVO_4_,^25^*etc.*, which have demonstrated the effectiveness of nano-composite methods for the photocatalytic activity improvement. Jiang prepared CuO/BiVO_4_ composite photocatalyst and found that the composite material showed the best photocatalytic performance when the loading amount of CuO was 2 wt%, achieving a methylene blue (MB) degradation rate of 47% in 180 min.^26^ Zhang prepared a new type of CuO–BiVO_4_ heterojunction composite material (CuO–BiVO_4_/FACS) by metal–organic decomposition impregnation method, and found that the composite material showed high photocatalytic activity for the degradation of MB under visible light irradiation, with a degradation rate up to 92% in 180 min.^27^ Zhao prepared carbon-loaded CuO–BiVO_4_ (CuO–BiVO_4_@C) composite photocatalytic materials by hydrothermal method, and CuO–BiVO_4_@1.0C showed the highest photocatalytic degradation activity for MB, with a degradation rate up to 100% in 2.5 h.^28^ Therefore, incorporating CuO into BiVO_4_ can effectively enhance the photocatalytic activity of pure BiVO_4_. However, to our knowledge, most CuO–BiVO_4_ composite materials have been studied for pollutant degradation, and there is still little research in the field of antibacterial applications. And there is a lack of in-depth analysis regarding the antibacterial mechanism of the CuO–BiVO_4_ composite materials.

Nano CuO is a low-cost, highly reactive broad-spectrum antibacterial material with advantages such as good heat resistance and stability. As an inorganic antibacterial material, it has good antibacterial performance without drug resistance, therefore, copper-based antibacterial materials have broad research and practical application prospects. It has been reported that needle-shaped nano CuO has strong antibacterial effects on *Escherichia coli* (*E. coli*) and *Staphylococcus aureus* (*S. aureus*).^29^ Ran immobilized CuO/BiVO_4_ photocatalytic materials on cotton fabric through a polydopamine template, achieving efficient visible light-driven photocatalysis, antibacterial, and ultraviolet protection applications.^30^ In this paper, a series of CuO/BiVO_4_ composite materials with different amounts of CuO loading were prepared by the hydrothermal-impregnation method. CuO/BiVO_4_ composite materials enhanced the visible-light response and reactive oxygen species (ROS) generation of BiVO_4_ for the photocatalytic antibacterial applications. Furthermore, the analysis of the antibacterial contribution of the CuO/BiVO_4_ composite materials successfully confirmed that photogenerated ROS serve as the primary mechanism, with superoxide anions (·O_2_^−^) and hydroxyl radicals (·OH) identified as the dominant reactive species. The process of bacterial inactivation by ROS was also elucidated.

## 2. Materials and methods

### 2.1 Materials

All reagents used in the experiments were of analytical grade, and all experiments were conducted with deionized water. All instruments used in the experiments were sterilized with an autoclave before use. Bismuth nitrate pentahydrate (Bi(NO_3_)_3_·5H_2_O) was obtained from Xilong Scientific Co., Ltd. Ammonium metavanadate (NH_4_VO_3_) was obtained from Tianjin Damao Chemical Reagent Factory (China). Dilute nitric acid, ammonia water, sodium dihydrogen phosphate and potassium dihydrogen phosphate were obtained from Tianjin Damao Chemical Reagent Factory (China). All biological reagents were purchased from Beijing Aoboxing Biotechnology Co., Ltd (China).

### 2.2 Synthesis of CuO/BiVO_4_ composite materials

Synthesis of BiVO_4_: 17.46 g of Bi(NO_3_)_3_·5H_2_O were dissolved in 80 mL of 2 mol per L dilute HNO_3_ to obtain a colorless transparent solution A. Then, 4.212 g of NH_4_VO_3_ were dissolved in 220 mL of 2 mol per L dilute HNO_3_ to obtain a yellow solution B. Solution A and B were mixed uniformly to obtain a yellow transparent solution and were stirred continuously for 0.5 h, then the pH was adjusted to 2 with ammonia to obtain an orange precipitate. After continue stirred at a constant speed for 1 h, the precipitate was aged at room temperature for 2 h. Then, the supernatant was poured off and about 80 mL of the slurry retained was transferred into a 100 mL Teflon-lined stainless steel autoclave, and heated at 200 °C for 24 h. After cooled to room temperature, the precipitate was washed three times with deionized water, dried under vacuum at 80 °C overnight, and then calcined in a muffle furnace at 400 °C for 2 h.

CuO/BiVO_4_ composite materials were prepared by impregnation-calcination method. Firstly, Cu(NO_3_)_2_ was dispersed in 20 mL of deionized water, and then the as-prepared BiVO_4_ was added to obtain a suspension. The suspension was stirred in a water bath at 80 °C until all the water evaporated. Finally, the remained powder was placed in a muffle furnace and calcined at 300 °C for 1 h. A series of composite materials were obtained by changing the amount of CuO loaded. The prepared composite materials were named as CuO/BiVO_4_-*x*% (*x* is the mass fraction of CuO in the CuO/BiVO_4_ composite material; *x* = 10, 12.5, 16.7, and 20).

### 2.3 Characterization

XRD measurement was conducted by Rigaku Ultima IV X-ray diffractometer from Japan. Copper target Cu-Kα radiation was used with wavelength of 1.5406 Å, divergence slit of 0.19 mm, tube voltage of 40 kV, and tube current of 40 mA. The scanning range was 10–80°, and the continuous scanning speed was 5° min^−1^. The surface morphology of samples was observed by Zeiss Gemini 300 scanning electron microscope from Germany. Before test, the samples were sputter-coated with gold to enhance the conductivity. The acceleration voltage was 5 kV, and the test mode was secondary electrons. XPS was performed by Shimadzu/Krayos AXIS Ultra DLD X-ray photoelectron spectrometer from Japan. Al-Kα (*hν* = 1486.6 eV) was used as the radiation source, and the power and pass energy of the spectrometer analyzer were 150 W and 50 eV, respectively. The electron binding energy was corrected based on the C 1s (284.8 eV) of the sample. The ultraviolet-visible spectra of the samples were tested by Shimadzu UV-3600i Plus spectrometer from Japan with the integrating sphere test mode. The wavelength range was 200–800 nm, and the diffuse reflectance (reflectance *R*%) data mode was selected. The copper ion concentration in the solution was tested by Agilent ICP-OES 725 ES with radio frequency power of 1.20 kW, flow rate of 15.0 L min^−1^, auxiliary flow rate of 1.50 L min^−1^, nebulizer flow rate of 0.75 L min^−1^, sampling delay of 10 s, replicate reading time of 15 s, and the number of repetitions of 3.

### 2.4 Photocatalytic activity test

#### 2.4.1 Inactivation of *E. coli*

The antibacterial performance of the CuO/BiVO_4_ composite materials was qualitatively measured by the colony counting method. 300 W xenon lamp with an ultraviolet light cutoff filter (*L*/420 nm) was used as the light source in the photocatalytic antibacterial experiment. The system temperature was maintained at around 25 °C during the antibacterial experiment by water cooling. All instruments and solutions required for the experiment were sterilized with high pressure, and the ultraviolet lamp in the sterile operation table was kept on 30 min before the experiment started. Firstly, 0.03 mg of CuO/BiVO_4_ composite material and 30 mL of sterilized PBS buffer solution were added to a 250 mL beaker, followed by the addition of *E. coli* suspension (10^6^ cfu mL^−1^). Before the photocatalytic antibacterial experiment, the CuO/BiVO_4_ composite material and bacterial suspension were stirred in the dark for 30 min. During the experiment, 0.1 mL of bacterial suspension was taken out every 10 min and transferred to the sterile operation table, diluted to an appropriate concentration, and then a suitable amount of suspension was dropped onto a Petri dish containing LB solid medium and spread.

After incubating the Petri dishes upside down in a constant temperature incubator for 24 h, the number of living cells (cfu) was counted. Dark control (no light) and blank control (no photocatalyst) experiments, as well as copper ion control experiments, were also conducted. All experiments were performed in triplicate, and the average values were given. The antibacterial rate *X* was calculated by eqn (1).1
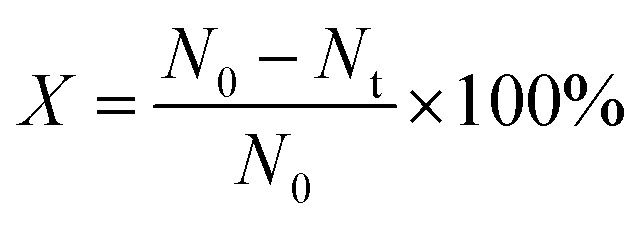
In the antibacterial experiments, *N*_0_ and *N*_t_ represent the number of viable cells in the blank control without CuO/BiVO_4_ and that after the photocatalytic reaction with the addition of CuO/BiVO_4_, respectively.

#### 2.4.2 Degradation of Rhodamine B

The photocatalytic degradation experiment of the CuO/BiVO_4_ composite materials was conducted under room temperature and visible light condition. A 300 W xenon lamp with an ultraviolet light cutoff filter (*L*/420 nm) was used as the light source. 100 mL of Rhodamine B solution (concentration of 10 mg L^−1^) was placed in a dry and clean beaker. The initial absorbance *A*_0_ was measured at wavelength of 554 nm. Then, the weighed CuO/BiVO_4_ composite materials were added to the beaker. After stirred in dark for 30 min, the absorbance was measured and recorded. Subsequently, the light source was turned on to initiate the photodegradation experiment. During the experiment, the absorbance of the solution was measured every 60 min. The absorbance recorded at different times was denoted as *A*_*t*_. The degradation efficiency *η* was calculated by eqn (2):2
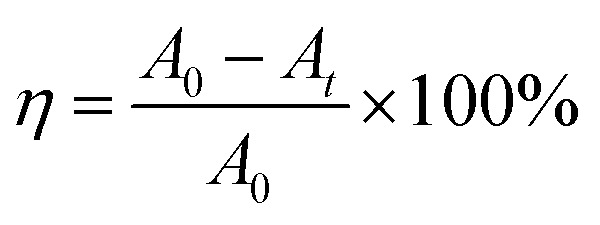


### 2.5 Determination of reactive oxygen species (ROS)

To analyze the free radicals, three reagents, superoxide dismutase (SOD), catalase (CAT), and d-mannitol were used to scavenge the ·O_2_^−^, H_2_O_2_, and ·OH radicals produced in the antibacterial process, respectively.^31^*E. coli* bacterial solution (10^6^ cfu mL^−1^) and CuO/BiVO_4_ composite materials (1000 µg mL^−1^) were placed into the beakers (Groups A, B, C), and then 300 µL of superoxide dismutase (SOD, 100 unit mL^−1^), 300 µL of catalase (CAT, 100 unit mL^−1^), and 300 µL of d-mannitol (10 mM) scavengers were added to the three beakers, respectively. The conditions and steps of the photocatalytic antibacterial experiment were the same as those in previous experiments. At the same time, control experiments with the scavengers alone in bacterial suspensions under visible light irradiation were also conducted. The influence of individual ROS species on the antibacterial performance of the CuO/BiVO_4_ composite material was analyzed by the colony counting method.

## 3. Results and discussion

### 3.1 Characterization of CuO/BiVO_4_ composite materials

**Fig. 1:**
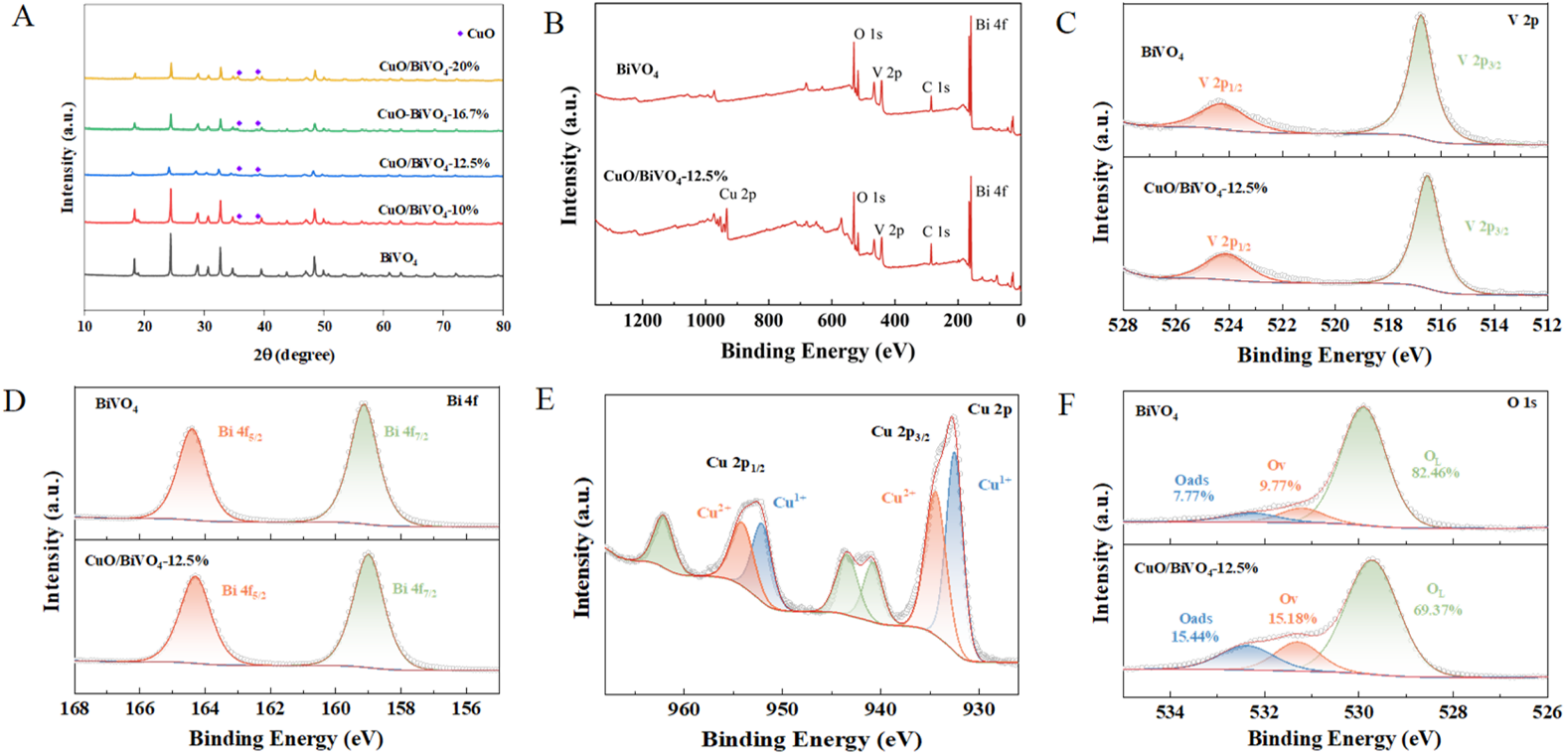
XRD patterns and XPS spectra of CuO/BiVO_4_ composite materials ((A) XRD; (B) total spectra; (C) V 2p; (D) Bi 4f; (E) Cu 2p; (F) O 1s).

To investigate the influence of CuO addition on the BiVO_4_ structure, the crystal structure of BiVO_4_ and CuO/BiVO_4_ composite materials was characterized by XRD. As shown in Fig. 1A, all the main characteristic peaks of pure BiVO_4_ matched with the monoclinic BiVO_4_ (m-BiVO_4_, JCPDS No. 14-0133), indicating the successful synthesis of monoclinic BiVO_4_. For the CuO/BiVO_4_ composite materials, the main characteristic peaks corresponded to those of pure BiVO_4_, and additional peaks observed at 35.6° and 38.7° were attributed to CuO (JCPDS No. 72-0629). The XRD diffraction peaks of pure CuO sample (Fig S1 in the SI) were consistent with those identified for CuO in the CuO/BiVO_4_ composite patterns presented in Fig. 1A, indicating the successful preparation of CuO/BiVO_4_ composite materials.

Further analysis of the elemental valence states and chemical composition of the CuO/BiVO_4_ composite materials was conducted by XPS. As shown in Fig. 1B, signals of Cu, V, Bi, O, and C were present in the composite materials. Fig. 1C–F were the high-resolution XPS spectra of V 2p, Bi 4f, Cu 2p, and O 1s, respectively. The high-resolution XPS spectra of V and Bi revealed that BiVO_4_ and CuO/BiVO_4_ were characterized by spin–orbit doublets. In the V 2p region (Fig. 1C), the peaks at around 516.5 eV and 524.0 eV were assigned to V 2p_3/2_ and V 2p_1/2_, respectively, signifying the V^5+^ oxidation state.^32^ In the Bi 4f region (Fig. 1D), the peaks located at approximately 159.0 eV and 164.1 eV were attributed to Bi 4f_7/2_ and Bi 4f_5/2_, respectively, characteristic of Bi^3+^.^34^ The XPS results confirmed the consistent oxidation states of the metal cations in materials. XPS analysis revealed that the Bi and V peaks in the CuO/BiVO_4_ composite shifted towards lower binding energies compared to the pristine BiVO_4_ sample. The peak corresponding to the Cu 2p_3/2_ orbital in Fig. 1E was deconvoluted, and the results suggested that there were Cu^2+^ and Cu^+^ in the composite materials.^33^ In Fig. 1F, O 1s binding energy was located at the range of 526.0 eV to 535.0 eV. The deconvolution results indicated that there were three types of oxygen in the composite materials, lattice oxygen, oxygen vacancy and adsorbed oxygen. Furthermore, the deconvolution of the O 1s spectra showed a notable increase in the proportion of oxygen vacancies, suggesting a higher concentration of surface oxygen vacancies in the CuO/BiVO_4_ composite materials.

**Fig. 2:**
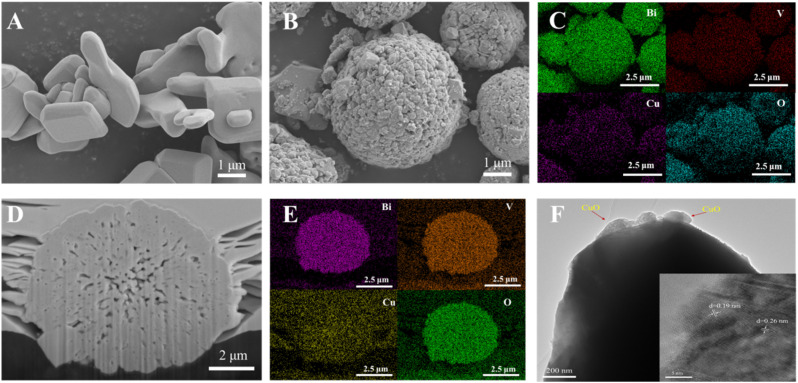
SEM/TEM images and EDS of CuO/BiVO_4_ composite materials ((A) pure BiVO_4_; (B) CuO/BiVO_4_-12.5%; (C) EDS mapping results; (D) cross-sectional SEM; (E) cross-sectional EDS mapping; (F) TEM and HRTEM).

SEM was conducted to explore the morphology of pure BiVO_4_ and CuO/BiVO_4_ composite materials. As shown in Fig. 2A, pure BiVO_4_ exhibited a relatively regular dodecahedral structure, with the length of 1.2 to 2.4 µm. The presence of some irregular structures may be due to incomplete growth of material. Fig. 2B and S2 in the SI displayed the morphology of the samples with CuO content increasing from 10 to 20%. The composite materials exhibited a relatively regular spherical appearance, which might originate from the recrystallization of BiVO_4_ in the acidic Cu(NO_3_)_2_ solution (pH of 3–4). Furthermore, EDS mapping confirmed the uniform distribution of Bi, V, Cu, and O elements on the surface of the composite materials (Fig. 2C). In order to determine the internal information of the particles, cross section polisher was used to cut the CuO/BiVO_4_ for further analysis by EDS. The cross-sectional SEM image (Fig. 2D) of the CuO/BiVO_4_ confirmed that there was no distinct interface between CuO and BiVO_4_ within the composite. Meanwhile, the cross-sectional EDS mapping (Fig. 2E) verified the uniform distribution of the Bi, V, Cu, and O elements inside the CuO/BiVO_4_ composite. Additionally, small CuO nanoparticles could be observed on the surface of the CuO/BiVO_4_ composite materials by TEM image (inset of Fig. 2F). The HRTEM image in Fig. 2F provided detailed investigation of the material's microstructure. Two types of lattice fringes with spacings of 0.26 nm and 0.19 nm were clearly observed, which could be assigned to the (111) plane of CuO and the (060) plane of BiVO_4_, respectively.

### 3.2 Photocatalytic antibacterial activity

**Fig. 3:**
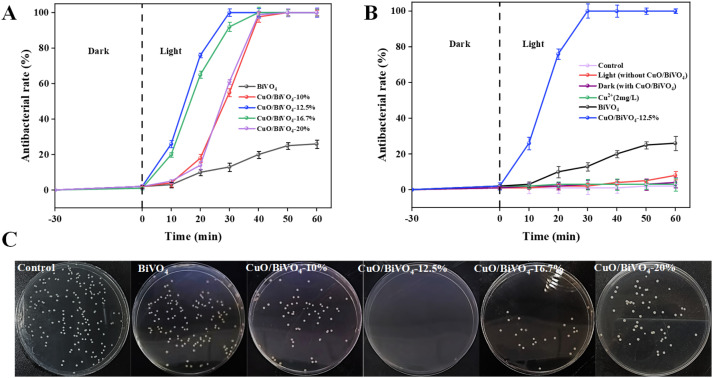
Antibacterial test and control experiment results of CuO/BiVO_4_ composite materials ((A) antibacterial test results; (B) control experiment results; (C) schematic diagram of antibacterial results).

The antibacterial performance of CuO/BiVO_4_ composite materials with CuO loading of 10%, 12.5%, 16.7%, and 20%, respectively, was evaluated by inactivation of *E. coli* under visible light. As shown in Fig. 3A, under visible light irradiation, CuO/BiVO_4_ composite materials exhibited good inactivation effects on *E. coli*, with antibacterial rate of 100% within 60 min. In contrast, the inactivation capability of pure BiVO_4_ was inferior, with an antibacterial rate of only 26% within 60 min. With the CuO content increased from 10% to 20%, the antibacterial rate of CuO/BiVO_4_ was 55%, 100%, 92%, and 61%, respectively, within 30 min. Therefore, 12.5% was selected as the optimal loading amount to prepare the CuO/BiVO_4_ composite materials. The antibacterial activity of CuO/BiVO_4_ in this study was comparable to that of recently reported BiVO_4_ based composite materials against *E. coli*, as shown in Table S1 in the SI. To exclude the influence of other factors on the antibacterial property, control experiments were conducted and the results were shown in Fig. 3B. In the dark control (with CuO/BiVO_4_) and blank control (light without CuO/BiVO_4_) experiments, the *E. coli* grew naturally, which indicated that the CuO/BiVO_4_ composite materials had no antibacterial ability in the absence of light, and the influence of visible light on bacteria was negligible. To exclude the influence of CuO on antibacterial performance, antibacterial tests were conducted on pure CuO. The results (Fig. S3 in the SI) showed that after 60 min of light exposure, the antibacterial rate of pure CuO was approximately 32%, indicating a negligible impact on antibacterial performance. Based on the ICP test, the concentration of Cu^2+^ released in solution was 1.35 mg L^−1^ after 60 min of illumination, and the inactivation effect of Cu^2+^ was insignificant.

**Fig. 4:**
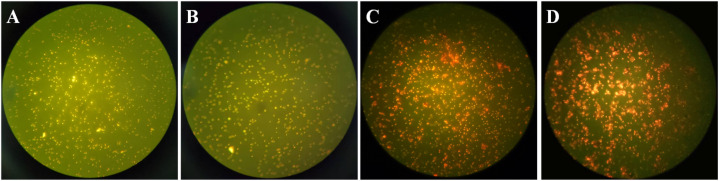
Fluorescence micrograph of CuO/BiVO_4_-12.5% for *E. coli* inactivation (A) negative control (B) dark for 30 min (C) light for 30 min (D) positive control.

Fluorescence microscopy can clearly observe the apoptosis of *E. coli* during the experimental process. According to the reagent manual, live bacterial cells stained with acridine orange (AO) reagent exhibit green fluorescence, while damaged or dead bacterial cells stained with propidium iodide (PI) reagent exhibit red fluorescence. As shown in Fig. 4, bacteria and composite material kept in dark for 30 min showed high-intensity green fluorescence, which was the same as the negative control (*E. coli* growing naturally), indicating that the dark adsorption process at the beginning of the experiment did not affect the growth of bacteria. While, all bacteria exhibited red fluorescence after 30 min of illumination, indicating that the composite material has completely inactivated the *E. coli* within the system.

**Fig. 5:**
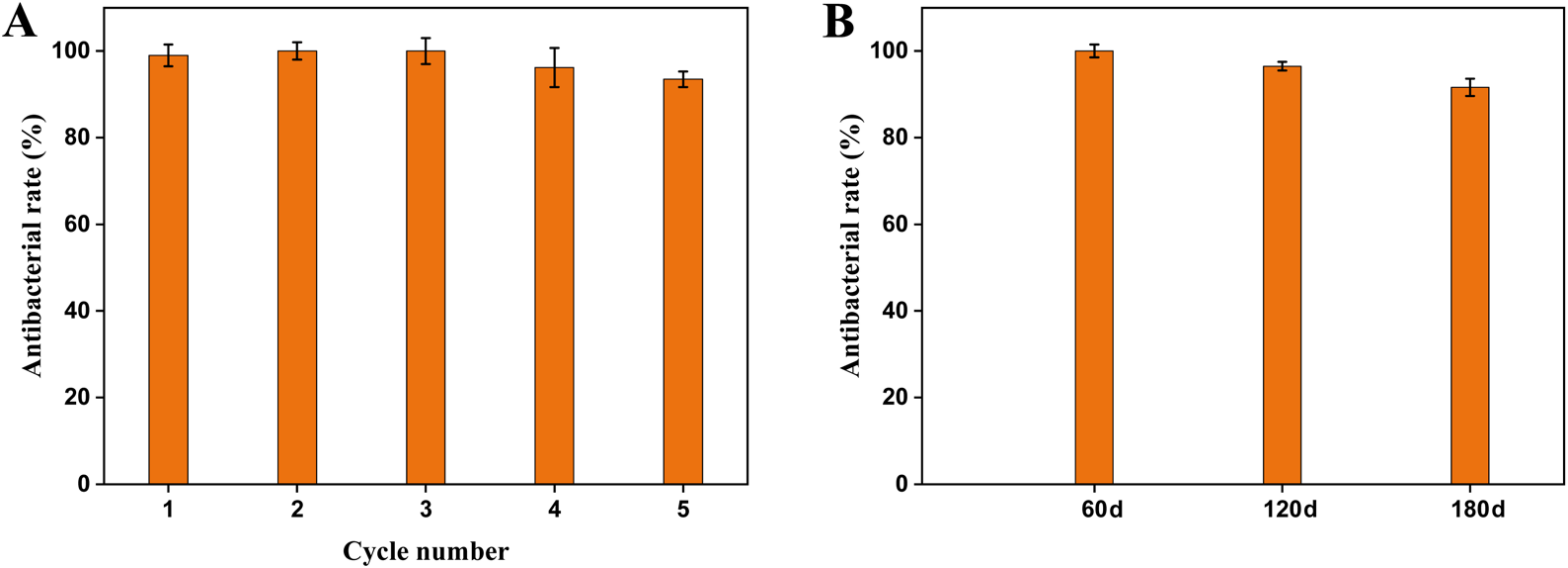
Cyclic and long-term experiments.

The cyclic and long-term antibacterial performance of the CuO/BiVO_4_-12.5% composite material against *E. coli* was presented in Fig. 5. After 5 cycles of antibacterial experiments, the antibacterial rate remained >90%, indicating that the composite material maintained good antibacterial activity and demonstrated excellent reusability and stability. The antibacterial rate of the composite material stored for 180 days was kept >90%, showing good long-term effectiveness. In summary, the CuO/BiVO_4_ composite material exhibited good reusability and stability, making it a promising visible light photocatalytic composite with practical application prospects.

**Fig. 6:**
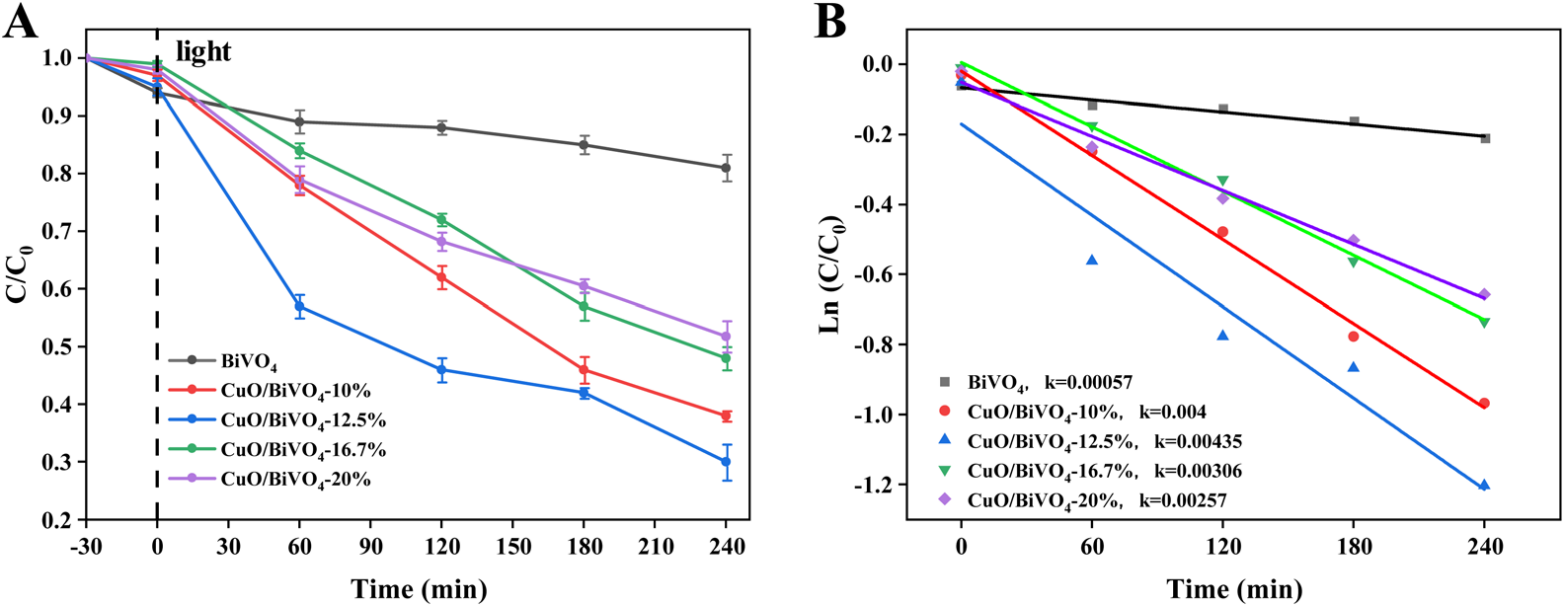
Photocatalytic degradation of Rhodamine B under visible light irradiation: (A) photodegradation rate; (B) dynamics curve.

To further explore the photocatalytic activity of the CuO/BiVO_4_ composite material, Rhodamine B was used as a model dye for degradation experiments. The initial concentration of Rhodamine B was 10 mg L^−1^ and the concentration of composite material was 1.0 g L^−1^. As shown in Fig. 6A, CuO/BiVO_4_ composite materials demonstrated good photocatalytic degradation performance under visible light irradiation, with much higher degradation rate than that of pure BiVO_4_. And the CuO/BiVO_4_-12.5% composite material showed the best photocatalytic degradation performance, achieving a degradation rate of 70% within 240 min, while the photocatalytic degradation performance of pure BiVO_4_ was relatively inferior, with a degradation rate of only 19%. The results indicated that the CuO loading could significantly enhance the photocatalytic activity of BiVO_4_. Fig. 6B is the first-order kinetic fitting curves for the degradation of Rhodamine B by the CuO/BiVO_4_ composite materials, and the *R*^2^ value (as shown in Table S2 in the SI) was close to 1, indicating a high degree of fitting. The slope of CuO/BiVO_4_-12.5% composite material was the largest, indicating the largest reaction constant and the highest photocatalytic activity. The dye degradation results further indicated that the significantly enhanced photocatalytic performance of CuO/BiVO_4_ composite material was originated from CuO loading.

**Fig. 7:**
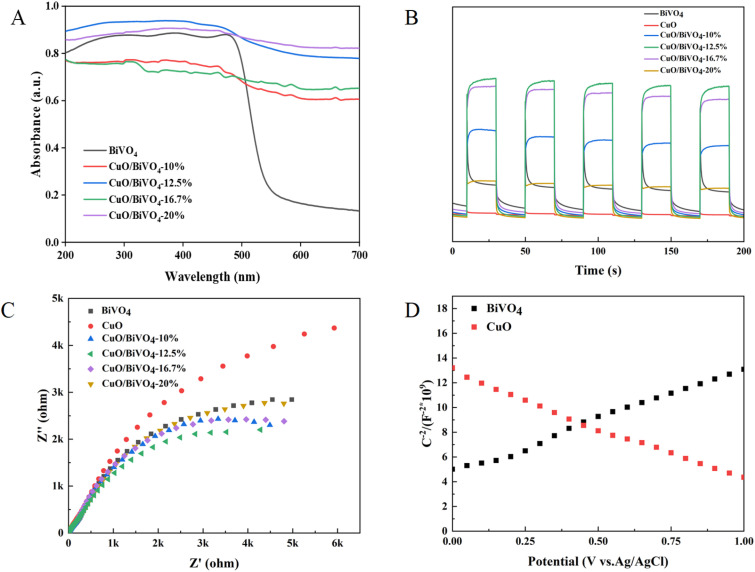
Characterization of the properties of the CuO/BiVO_4_ composite: (A) UV-Vis diffuse reflectance spectra (DRS), (B) photocurrent density, (C) EIS Nyquist plots, (D) Mott–Schottky plots.

UV-Vis diffuse reflectance spectroscopy (DRS) was used to study the optical absorption properties of the prepared materials. As shown in Fig. 7A, the visible light absorption edge of BiVO_4_ was at 562 nm. In contrast, the visible light absorption edge of CuO/BiVO_4_ extended to approximately 611 nm, with obvious absorption of visible light. The results demonstrated that CuO/BiVO_4_ possessed a broader visible light response range and higher visible light utilization efficiency than BiVO_4_. Electrochemical measurements were performed to evaluate the photocurrent density and charge transfer efficiency of CuO/BiVO_4_. As shown in Fig. 7B, the CuO/BiVO_4_ composite exhibited a significantly higher transient photocurrent density than pure BiVO_4_. The enhanced photocurrent response indicated a more efficient separation of photogenerated electron–hole pairs. To probe the interfacial charge mobility within the photocatalyst, electrochemical impedance spectroscopy (EIS) was employed (Fig. 7C). The Nyquist plot of the CuO/BiVO_4_ composite showed a markedly smaller arc radius compared to that of pure BiVO_4_, suggesting a lower charge transfer resistance. The reduction in resistance was conducive to more efficient charge migration, which was consistent with the superior photoresponse performance observed for the CuO/BiVO_4_ composite materials. The Mott–Schottky measurements (Fig. 7D) identified CuO as p-type and BiVO_4_ as n-type semiconductors. The results further confirmed the superior photoelectrochemical performance of CuO/BiVO_4_ composite materials compared with BiVO_4_.

### 3.3 Photocatalytic antibacterial mechanism

**Fig. 8:**
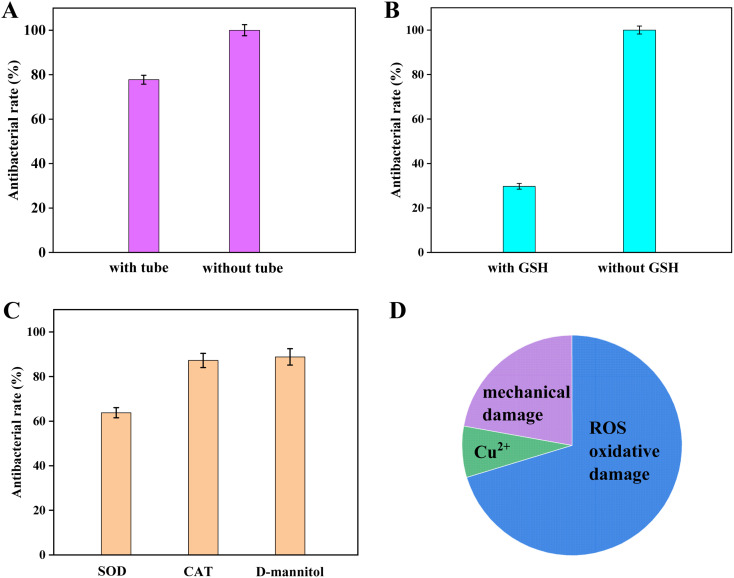
Antibacterial activity ((A) dialysis tube experiment; (B) GSH experiment; (C) single ROS scavenger experiment) and the contribution of each antibacterial mechanism.

Currently, there are three main antibacterial mechanisms for inorganic photocatalytic materials: ROS oxidative damage mechanism, ion leaching mechanism, and mechanical damage mechanism.^35^ Herein, the photocatalytic antibacterial mechanism of the CuO/BiVO_4_ composite material was explored. Firstly, the dialysis tube experiment was determined. When the CuO/BiVO_4_ composite material was placed inside the dialysis tube, ROS and leached ions could diffuse into the bacterial solution through the dialysis membrane, while the CuO/BiVO_4_ was confined within the dialysis without direct contact with the bacteria, thus preventing mechanical damage of bacteria. As shown in Fig. 8A, the antibacterial rate of CuO/BiVO_4_ was 100% without dialysis tube. However, when the composite material was confined within the dialysis tube, the antibacterial rate reduced to 77.8%. The result indicated that the ROS oxidative and the ion leaching damage mechanism showed main contribution to the photocatalytic antibacterial mechanism of CuO/BiVO_4_. In order to estimate the effect of ROS, glutathione (GSH, ROS scavenger) was added to the system, and the results were shown in Fig. 8B. After the addition of GSH, the antibacterial rate decreased to 29.7%, which indicated that the ROS oxidation damage contributed 70.3% to the antibacterial mechanism of the composite material. Therefore, the antibacterial contribution of Cu^2+^ was *ca.* 7.5%, consistent with the insignificant effect of Cu^2+^ in Fig. 3B. Based on the above results, ROS oxidative damage is the primary mechanism of CuO/BiVO_4_ composite material, with the mechanical damage and leaching of Cu^2+^ playing a synergistic role (Fig. 8D).

To further clarify the contribution of specific free radicals to the antibacterial activity of the CuO/BiVO_4_ composite material, single ROS scavenging test was conducted. Superoxide dismutase (SOD), catalase (CAT), and d-mannitol can respectively scavenge ·O_2_^−^, H_2_O_2_, and ·OH, and the three scavengers are specific and do not possess antibacterial capabilities themselves.^36^ As shown in Fig. 8C, after the addition of SOD, CAT, and d-mannitol, the antibacterial rates of CuO/BiVO_4_ decreased to 63.8%, 87.2%, and 88.8%, respectively. The results indicated that during the antibacterial process of the CuO/BiVO_4_ composite material, ·O_2_^−^ played a major role, while H_2_O_2_ and ·OH played a secondary role. The EPR results further confirmed that CuO/BiVO_4_ could produce more ·O_2_^−^ compared to pure BiVO_4_ under light excitation (Fig. S4 in the SI).

**Scheme 1:**
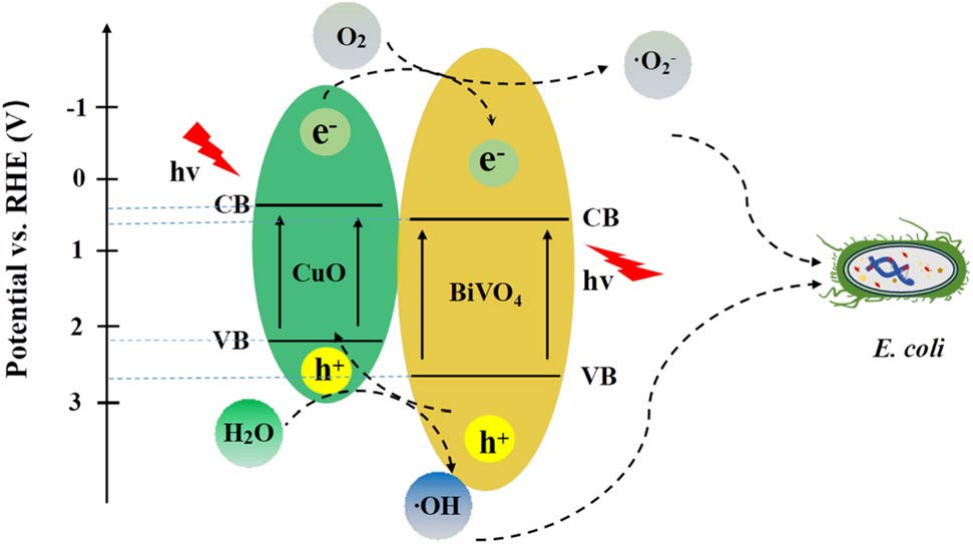
Photocatalytic antibacterial mechanism of the CuO/BiVO_4_ composite material under visible light irradiation.

According to the antibacterial results and photoelectrochemical test, we propose a photocatalytic antibacterial mechanism of CuO/BiVO_4_ composite material (Scheme 1). The detailed calculation of CB, VB, and bandgap for BiVO_4_ was shown in the SI (Fig. S5). Under visible light irradiation, photogenerated electrons (e^−^) on the conduction band of the p-type CuO are transferred to the conduction band of the n-type BiVO_4_, meanwhile, holes (h^+^) on the valence band of the n-type BiVO_4_ move to the valence band of the p-type CuO. In this way, the photogenerated e^−^–h^+^ pairs can be quickly and effectively separated, thus significantly reducing the recombination of e^−^–h^+^ pairs and exhibiting improved photocatalytic performance. Subsequently, the photogenerated electrons (e^−^) and holes (h^+^) migrated to the surface and underwent redox reactions with adsorbed O_2_ and H_2_O molecules, generating reactive oxygen species (ROS) with strong oxidizing capabilities, such as superoxide radicals (·O_2_^−^) and hydroxyl radicals (·OH). These reactive substances will undergo oxidation reactions with proteins, nucleic acids, and cell membranes in bacterial cells, disrupting the normal growth and reproduction of bacteria and finally killing the bacteria.

## 4. Conclusion

In summary, the CuO/BiVO_4_ composite photocatalyst was successfully prepared by hydrothermal-impregnation method. CuO loading onto BiVO_4_ was beneficial for the separation and transfer of photogenerated electrons (e^−^) and holes (h^+^), consequently, the photocatalytic antibacterial activity was significantly enhanced compared to pure BiVO_4_. The CuO/BiVO_4_-12.5% composite demonstrated the best bactericidal activity, achieving a 100% antibacterial rate under visible light irradiation within 30 min. The outstanding antibacterial performance is mainly originated from ROS oxidative damage, in which ·O_2_^−^ played a major role in antibacterial activity. The CuO/BiVO_4_ composite material also exhibited good stability for repeated use. The performance advantages and scalable synthesis methods of CuO/BiVO_4_ composites make them highly promising for antibacterial applications.

## Conflicts of interest

There are no conflicts to declare.

## Supplementary Material

RA-015-D5RA06653K-s001

## Data Availability

All data supporting this study are provided in the manuscript and supplementary information (SI) is available from the corresponding author upon reasonable request. Supplementary information: XRD pattern and antibacterial performance of CuO, the SEM images of CuO/BiVO_4_ composite materials, the EPR spectra of ·O_2_^−^, the band gap and VB/CB potential calculation, and slope and *R*^2^ value of dynamic fitting curve. See DOI: https://doi.org/10.1039/d5ra06653k.
