# Supplementary material for: Fabrication of CuO/BiVO4 composites for enhanced visible-light-driven photocatalytic antibacterial activity
Source: RSC Adv. 2025 Nov 18;15(53):45038–47. doi: 10.1039/d5ra06653k (PMC12625661; doi:10.1039/d5ra06653k)
Supplement: RA-015-D5RA06653K-s001 [file RA-015-D5RA06653K-s001.pdf]

Supplementary Information (SI) for RSC Advances.
This journal is © The Royal Society of Chemistry 2025

Supplementary Materials

Fabrication of $CuO/BiVO_4$ composites for enhanced visible-light-driven photocatalytic antibacterial activity

Jiao Zhao*, He Yang, Chen Chen, Tong You, Xingyi Yu, Yan Zhang, Hongyan Liu, and Yimin Zhu*

Collaborative Innovation Center for Vessel Pollution Monitoring and Control, Dalian Maritime University, Dalian 116026, China.

* Corresponding E-mail: zhaojiao@dlmu.edu.cn, ntp@dlmu.edu.cn

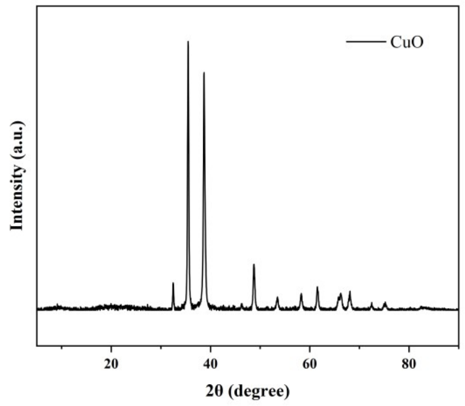

Figure S1. XRD pattern of CuO

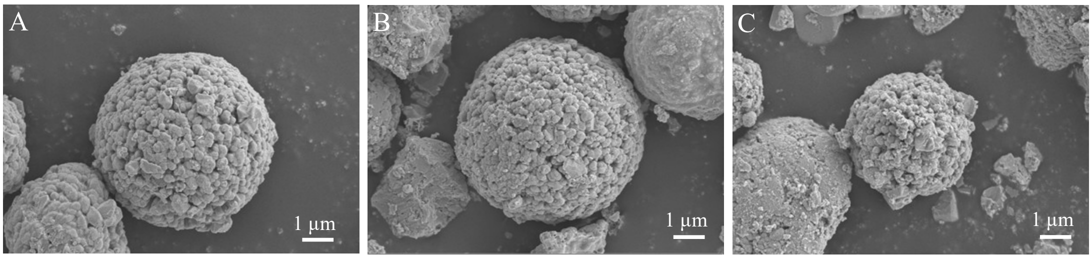

Figure S2. SEM images of $CuO/BiVO_4$ composite materials (A: $CuO/BiVO_4$-10%; B: $CuO/BiVO_4$-16.7%; C: $CuO/BiVO_4$-20%)

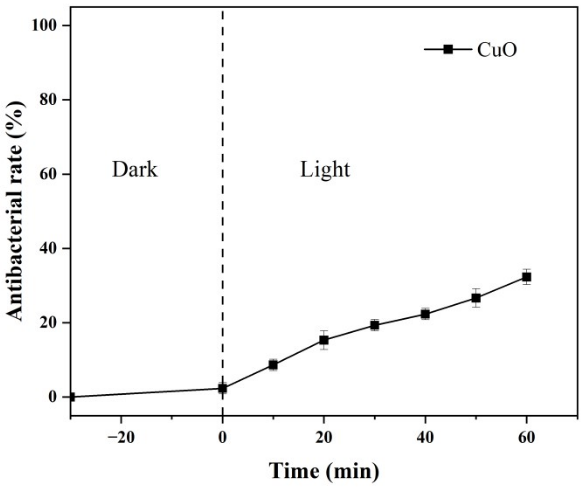

Figure S3. Antibacterial performance of CuO

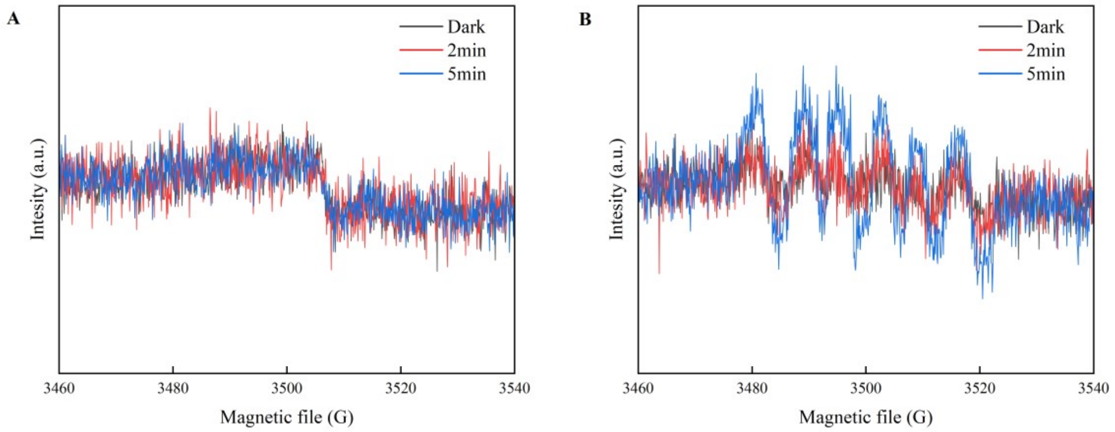

Figure S4. EPR spectra of $\cdot O_2^-$ produced by (A) $BiVO_4$ and (B) $CuO/BiVO_4$

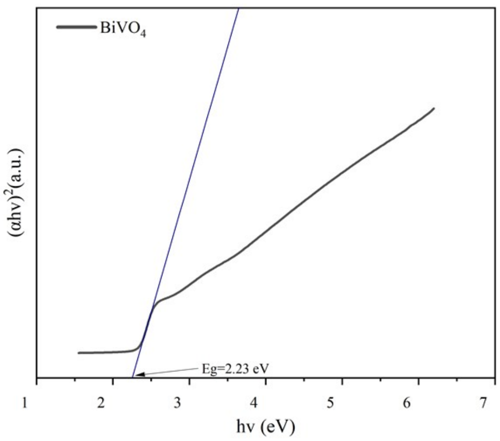

Figure S5. UV-Vis spectrum of $BiVO_4$: relationship between $(\alpha hv)^2$ and hv

Based on the literature, the band gap of CuO was 1.7 eV, with the VB potential of 2.16 eV and CB potential of 0.46 eV[1].

Based on the UV–vis DRS results, the band gap value of $BiVO_4$ was calculated by Equation (1), and a Tauc plot was constructed.

$$(\alpha hv)^n = A(hv - Eg) \quad (1)$$

Where A, Eg, α, h, and v denote the constant, band gap energy, absorption coefficient, Planck constant, and optical frequency, respectively. The value of n depends on the type of electronic transition in the semiconductor: n=2 for semiconductors with direct allowed transitions and n=1/2 for those with indirect allowed transitions. Since $BiVO_4$ is a semiconductor with direct allowed electronic transitions, Eg can be derived from the intercept of the $(\alpha hv)^2$ curve on the x-axis. It can be seen from Figure S5 that the Eg value of $BiVO_4$ is 2.23 eV. The conduction band edge (E_{CB}) and valence band edge (E_{VB}) of a semiconductor can be calculated using the Equations (2-3)[2].

$$E_{CB}=\mathrm{X}-E_C-1/2Eg \quad (2)$$

$$E_{VB}=E_{CB}+Eg \quad (3)$$

Where X is the absolute electronegativity of the atom semiconductor; E_C is the energy of free electrons of the hydrogen scale (4.5 eV); Eg is the band gap of semiconductor; and E_{VB} is the VB edge potential and the E_{CB} is the CB edge potential.

It can be calculated that the valence band edge (E_{VB}) of $BiVO_4$ is 2.65 eV and the conduction band edge (E_{CB}) of $BiVO_4$ is 0.42 eV.

Table S1 Antibacterial performance of $BiVO_4$ based nanomaterials

Materials	Preparation method	Concentration (μg/mL)	*E. coli* (CFU/mL)	Antibacterial rate	Ref.
$V_2O_5/BiVO_4$	Precipitation	1000	10^8	99%	[3]
$BiO_4/Bi_2WO_6/WO_3$	Hydrothermal	1000	10^6	100%	[4]
$BiVO_4$/g-C_3N_4	In situ solid-state	800	10^7	99%	[5]
Ag/$BiVO_4$	Hydrothermal	1200	10^6	100%	[6]
Fe-doped $BiVO_4$	Sol-Gel	1000	10^6	100%	[7]
Biochar/$BiVO_4$	Impregnation-calcination	900	10^6	99.5%	[8]
AgI/$BiVO_4$	Precipitation-photor eduction	1000	10^6	99.88%	[9]
CuO/$BiVO_4$	Impregnation-calcination	1000	10^6	100%	This work

Table S2 Slope and R^2 value of dynamic fitting curve

	$BiVO_4$	CuO/$BiVO_4$-10%	CuO/$BiVO_4$-12.5%	CuO/$BiVO_4$-16.7%	CuO/$BiVO_4$-20%
k (min^{-1})	0.00057	0.004	0.00435	0.00306	0.00257
R^2	0.96604	0.99618	0.93699	0.99508	0.98899

References

[1] Y. Kanigaridou, A. Petala, Z. Frontistis, M. Antonopoulou, M. Solakidou, I. Konstantinou, D. I. Kondarides. Solar photocatalytic degradation of bisphenol A with $CuO_x/BiVO_4$: insights into the unexpectedly favorable effect of bicarbonates[J]. Chemical Engineering Journal, 318 (2017) 39-49.

[2] H. Xu, H. Li, C. Wu, J. Chu, Y. Yan, H. Shu, Z. Gu, Preparation, characterization and photocatalytic properties of Cu-loaded BiVO4[J]. Journal of Hazardous Material, 153 (1-2) (2008)877-884.

[3] Wang Y, Long Y, Zhang D. Novel bifunctional $V_2O_5/BiVO_4$ nanocomposite materials with enhanced antibacterial activity[J]. Journal of the Taiwan Institute of Chemical Engineers, 2016, 68: 387-395.

[4] Cho E-C, Hsiao Y-S, Huang J-H, et al. Construction of $BiVO_4/Bi_2WO_6/WO_3$ heterojunctions with improved photocatalytic capability in elimination of dye and antibiotics and inactivation of E. coli[J]. Journal of the Taiwan Institute of Chemical Engineers, 2023, 149: 104991.

[5] Khan A, Altaf M, Shahid M, et al. In situ solid-state fabrication of Z-Scheme $BiVO_4/g\text{-}C_3N_4$ heterojunction photocatalyst with highly efficient-light visible activity and their antibacterial properties against bacterial pathogens[J]. Journal of Molecular Structure, 2024, 1300: 137222.

[6] Regmi C, Dhakal D, Lee S W. Visible-light-induced $Ag/BiVO_4$ semiconductor with enhanced photocatalytic and antibacterial performance[J]. Nanotechnology, 2018, 29(6): 064001.

[7] Regmi C, Kshetri Y K, Kim T-H, et al. Visible-light-induced Fe-doped $BiVO_4$ photocatalyst for contaminated water treatment[J]. Molecular Catalysis, 2017, 432: 220-231.

[8] Wang T, Cai J, Zheng J, et al. Facile synthesis of activated biochar/$BiVO_4$ heterojunction photocatalyst to enhance visible light efficient degradation for dye and antibiotics: applications and mechanisms[J]. Journal of Materials Research and Technology, 2022, 19: 5017-5036.

[9] Xiang Z, Wang Y, Ju P, et al. Facile fabrication of $AgI/BiVO_4$ composites with enhanced visible photocatalytic degradation and antibacterial ability[J]. Journal of Alloys and Compounds, 2017, 721: 622-627.
